# The Synthesis of Well-Dispersed and Uniform-Sized Zeolite NaY by Adding Non-Refluxed and Acid-Refluxed Cogon Grass

**DOI:** 10.3390/ma16237330

**Published:** 2023-11-24

**Authors:** Pakawan Sereerattanakorn, Pimwipa Tayraukham, Nattawut Osakoo, Panot Krukkratoke, Chalermpan Keawkumay, Jatuporn Wittayakun, Nichakorn Pornnongsan, Krittanun Deekamwong, Sanchai Prayoonpokarach

**Affiliations:** 1 School of Chemistry, Institute of Science, Suranaree University of Technology, Nakhon Ratchasima 30000, Thailand; 2 Institute of Research and Development, Suranaree University of Technology, Nakhon Ratchasima 30000, Thailand

**Keywords:** zeolite NaY, cogon grass, paraquat adsorption, acid-refluxed, uniform crystal size

## Abstract

Zeolite NaY synthesized from a typical procedure could suffer from agglomeration. Adding non-refluxed cogon grass (NG) to the synthesis gel could produce NaY with good dispersion and uniform crystal size. Small molecules produced from cogon grass in alkaline conditions could prevent agglomeration. The obtained zeolite (Y-NG) has a crystallinity and surface area comparable to the synthesis without grass (Y-WG). Y-NG demonstrated similar paraquat adsorption capacity to Y-WG at high initial concentrations. On the other hand, the zeolite from the addition of acid-refluxed grass (Y-RG) has the lowest crystallinity, smallest surface area, and poorest paraquat adsorption capacity. The effect of grass amount on the zeolite structure was studied. One gram of cogon grass was the optimum amount to add to the synthesis gel.

## 1. Introduction

Zeolite NaY is a member of the faujasite framework (FAU), with a typical silicon-to-aluminum ratio (Si/Al) in the range of 2–5 and sodium as a charge-balancing ion [1]. NaY has three-dimensional channels with a pore diameter of 7.4 Å. Its outstanding properties, such as a large surface area and high sodium content, have made it versatile for various applications, such as an adsorbent of paraquat and dye [2,3,4]. There are still challenges in the synthesis of zeolite NaY to achieve precise control over its properties for specific applications such as crystal size, structure, and chemical composition [5]. The properties of NaY depend on various synthesis parameters [5,6].

Efforts have been made in synthesis methods to optimize the characteristics of zeolites. One approach is the use of additives in one-pot synthesis, which allows for tailoring the properties of zeolites [7,8]. Additives play a crucial role in inducing the aggregation of precursor molecules and facilitating zeolite particle nucleation [9]. For instance, researchers have explored the utilization of biomass as an additive in zeolite synthesis. Sugarcane bagasse residue was investigated to produce a new morphology in MFI zeolite [10,11,12]. Biomass-derived additives can impact the direction of crystal growth and zeolite formation. Hydrolysis of bagasse under base conditions generates molecules comprising both hydrophilic sites (hydroxyl groups) and hydrophobic sites (organic species) [13,14]. The hydroxyl groups have shown potential as binding sites for zeolite precursors [13,14]. In related studies, glucose was employed in the synthesis of various hierarchical ZSM-5 materials [15]. The preference for the interaction between hydrocarbon molecules and aluminates over silicates leads to a higher aluminum content in the final product [16,17]. These findings highlight the influence of additives on the chemical composition and properties of zeolites, showcasing the potential of biomass-derived additives in zeolite synthesis.

An interesting biomass in this work is cogon grass (*Imperata cylindrica*) because it is abundant worldwide but has low value. Cogon grass is an invasive species widely regarded as undesirable due to its aggressive growth, high flammability, and ability to outcompete native plant species. It quickly spreads, forming dense stands and negatively impacting biodiversity [18,19]. The grass poses a fire hazard and can reduce agricultural productivity by invading fields and interfering with crop growth. Additionally, it has little nutritional value for livestock and can disrupt grazing areas [20]. Efforts to control cogon grass face challenges due to its resilience and rapid recolonization. Due to its abundance, researchers have been finding ways to increase its value. With a silica content of around 9% in dry weight, cogon grass was used as a silica source to synthesize zeolite NaY and NaX [4,21]. The organic content in cogon grass could have an effect as a stabilizing agent in zeolite synthesis.

The compositions of cogon grass by weight are cellulose (35.1%), hemicellulose (27.6%), lignin (16.5%), and extractives (11.2%) [22]. When sodium hydroxide is mixed with cogon grass, it reacts with the ester bonds in the cellulose and other organic compounds of the cogon grass, converting them into smaller carbohydrate molecules, alcohol, and sodium salt. Those products could influence zeolite formation and crystallization. Regarding bio-silica from cogon grass, acid leaching decomposes organic components and removes inorganic components, resulting in higher-purity silica [21,23].

Therefore, this work aims to utilize cogon grass to synthesize zeolite NaY. Non-refluxed and acid-refluxed grass are compared to understand the role of organic contents produced in situ within the synthesis gel. Characterization techniques, including X-ray diffraction (XRD), Fourier transform infrared spectroscopy (FTIR), and scanning electron microscopy with energy dispersive spectroscopy (SEM-EDS) are employed to distinguish the properties of the obtained zeolites. Moreover, the adsorption of the herbicide paraquat is used as the first indicator to differentiate the zeolite samples. Previous works have reported that zeolite with different properties has different capacities for paraquat adsorption. The adsorption on zeolite NaY and NaBEA obeys the Langmuir model, with NaY as a better adsorbent [2]. Further studies have found that NaY has a higher adsorption capacity than NaX due to a higher Si/Al ratio [24], and the adsorption on NaY increased with the Si/Al ratio [3].

## 2. Materials and Methods

### 2.1. Materials

Cogon grass was collected from the Suranaree University of Technology (Nakhon Ratchasima, Thailand). The chemicals used were hydrochloric acid (37%, Merck, Rahway, NY, USA), silicon dioxide RPE (99%, Sigma-Aldrich, St. Louis, MO, USA), sodium aluminate (95%, Sigma-Aldrich), and sodium hydroxide (97%, Carlo Erba, Emmendingen, Germany). Commercial-grade paraquat (N,N′-dimethyl-4,4′-bipyridinium dichloride, 27.6%, Shandong Kexin Biochemical, Jinan, China) was used in the adsorption studies. Sodium silicate solution (Na_2_SiO_3_) was prepared by dissolving 28.7 g of fumed silica in 59.8 mL of 0.27 M NaOH solution.

### 2.2. Preparation of Cogon Grass

Cogon grass was washed with water, cut to 0.5 cm in length, and dried in a hot air oven at 90 °C for 3 days. This non-refluxed grass was named NG. The 5.0 g of dried NG was refluxed by using 67.0 mL of 3.0 M HCl solution at 90 °C for 6 h. The solid portion was filtered, washed with deionized (DI) water until pH neutral, and dried at 90 °C for 24 h. The refluxed grass is named RG.

### 2.3. Synthesis of Zeolite NaY by Adding Cogon Grass

Zeolite NaY was synthesized with a process modified from that of the Synthesis Commission of the International Zeolite Association [25]. The molar composition of the overall gel used was 1Al_2_O_3_:10SiO_2_:5.1Na_2_O:180H_2_O. Typically, 1.0 g of dried NG or RG was soaked with 10.0 mL of 2.5 M NaOH at ambient temperature for 24 h. These samples were named NGS and RGS. The mixture was transferred into a 250 mL polypropylene bottle and added to 4.8 g of Na_2_SiO_3_ solution and 0.4 g of NaAlO_2_ under continuous stirring. The resultant mixture, a seed gel, was aged at room temperature for 24 h.

A feed gel was prepared similarly to a seed gel without aging. Briefly, 2.8 g of NaAlO_2_ and 30.18 g of Na_2_SiO_3_ solution were added to NaOH solution containing 0.035 g of NaOH in 22.3 mL of DI water. After that, a feed gel was gradually dropped into the seed gel with stirring and continuously stirred for 10 min. The final gel was aged at ambient temperature and crystallized at 90 °C for 24 h. The resulting mixture was washed with DI water until the measured pH was neutral by centrifugation and dried at 90 °C for 24 h. The zeolite samples from non-refluxed and refluxed grass were designated as Y-NG and Y-RG, respectively. The same method was used to produce parent NaY zeolite without any grass. The sample was named Y-WG. The sample Y-NG was also called Y-1.0NG to compare the grass amounts (Section 2.6). All samples were not calcined.

### 2.4. Paraquat Adsorption on Zeolite Samples

The paraquat adsorption was performed as described in the literature [4], using paraquat concentrations ranging from 100 to 1000 mg/L. The adsorption from each concentration was repeated three times. In each experiment, 0.05 g of dried NaY sample was added to 20 mL of the desired concentration of paraquat solution under magnetic stirring (450 rpm) for 60 min at 30 °C with a water bath-controlled temperature. The remaining paraquat solution was separated through a 0.45 µm syringe filter and analyzed by a UV-Vis spectrophotometer (Varian CARY 300) at $\lambda_{max}$ of 257 nm. The calibration curve of paraquat solution is shown in Figure S1 in Supplementary Materials. The paraquat adsorption data were evaluated with Langmuir and Freundlich isotherm models.

The maximum adsorption capacity of paraquat (*q_m_*) on absorbents was determined based on the Langmuir isotherm using Equation (1).

(1) $$\frac{C_{e}}{q_{e}}=\frac{1}{K_{L}q_{m}}+\frac{C_{e}}{q_{m}}$$

where *C_e_* and *q_e_* are the concentration and amount adsorbed at equilibrium, and *K_L_* is the Langmuir constant related to the affinity of the binding site (L/mg). Both *K_L_* and *q_m_* can be determined from the linear plot of *C_e_*/*q_e_* versus *C_e_*.

The Freundlich adsorption equation is defined based on Equation (2).

(2) $$log\;q_{e}=log\;K_{F}+\frac{1}{n}log\;C\;$$

where *K_F_* is the Freundlich constant, which indicates the relative adsorption capacity of adsorption (mg/g) to proceed in multilayer sorption, while 1/*n* represents adsorption intensity.

### 2.5. Characterization of Zeolite Samples

Phases of the samples were confirmed by X-ray diffraction (XRD, Bruker D8 ADVANCE, Billerica, MA, USA) with Cu Kα radiation operated at 40 kV and 40 mA. Relative crystallinity was calculated from the XRD data. The total area of nine prominent peaks of FAU zeolite was determined by OriginLab, Origin 2022 (9.9). Y-NG has the highest total area and was used as a reference. The functional groups of the samples were investigated by Fourier-transform infrared spectroscopy (FT-IR, Bruker Tensor 27) using attenuated total reflectance (ATR) mode. The morphology, particle size, and Si/Al ratio of the zeolite products were determined by field-emission scanning electron microscopy with energy-dispersive X-ray spectroscopy (FE-SEM/EDX, JEOL JSM 7800F, Akishima, Tokyo, Japan). The samples were dispersed on carbon tape and coated with gold for 1 min to increase their conductivity. Surface areas were determined by N_2_ adsorption/desorption using the Brunauer–Emmett–Teller (BET) method and the *t*-plot method. Thermal decomposition of the products was studied by thermogravimetric analysis (TGA, Mettler Toledo TGA/DSC1, Schwerzenbach, Switzerland) in the air-zero with a flow rate of 50.0 mL/min at a heating rate of 10.0 °C/min up to 700 °C.

### 2.6. Synthesis and Characterization of Zeolite NaY with Various Amounts of Non-Refluxed Cogon Grass

Zeolite NaY was synthesized with the procedure described in Section 2.3, but with 0.5, 1.5, and 2.0 g of non-refluxed cogon grass. The obtained products were named Y-0.5NG, Y-1.5NG, and Y-2.0NG, respectively. They were characterized by XRD, SEM, and TGA and compared with Y-1.0NG from Section 2.3.

### 2.7. Effect of Calcination on Adsorption Capacity

The synthesis of Y-NG was repeated twice with 1.0 g of non-refluxed grass from the same location. One gram of each zeolite product was calcined at 500 °C for 3 h with a heating rate of 1 °C/min. Before testing for paraquat adsorption, all non-calcined and calcined samples were characterized by XRD (Bruker D2 PHASER) and tungsten scanning electron microscopy (W-SEM, JEOL, JSM-6010LV).

## 3. Results and Discussion

### 3.1. Paraquat Adsorption on the Zeolite Samples

**Figure 1:**
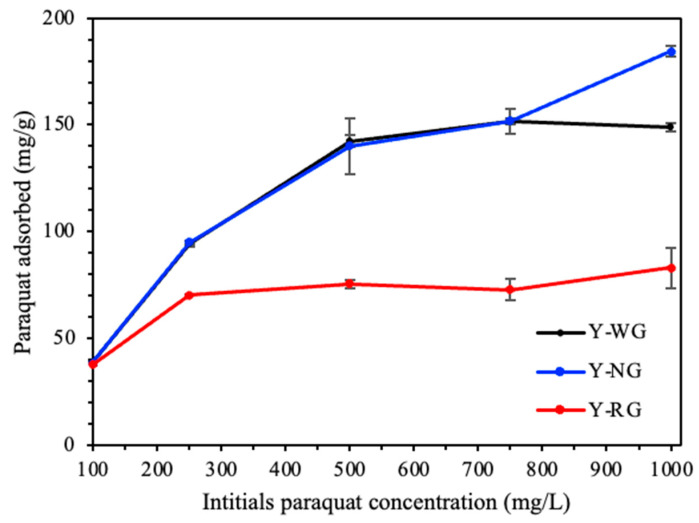
Adsorption of paraquat on Y-WG, Y-NG, and Y-RG.

Figure 1 shows the plot between the paraquat adsorbed versus the initial concentration of NaY synthesized using refluxed grass (Y-RG), non-refluxed grass (Y-NG), and the conventional synthesis method without grass (Y-WG) under standard conditions. Table 1 lists the parameters and correlation coefficients (R^2^) from fitting paraquat adsorption on the zeolite samples with the Langmuir (see the data in Table S1 in Supplementary Materials) and Freundlich models (see details in Figure S2 in Supplementary Materials). The adsorption on all adsorbents fits well with the Langmuir isotherm, indicating that the adsorption sites are uniform, and the monolayer is formed. Y-WG exhibits the best fit, with an R^2^ higher than Y-NG and Y-RG. An excellent fit was also reported on zeolite NaY and NaBEA [2,24]. At 1000 mg/L, the adsorption on Y-WG was essentially constant, whereas that on Y-NG and Y-RG increased. This was likely due to multilayer adsorption. Consequently, Y-NG has a higher adsorption capacity than Y-WG as shown in Table 1, and more details are provided in Table S2 in Supplementary Materials. From the fit with the Freundlich models (Table 1), Y-NG and Y-RG had higher R^2^ values than Y-WG. It was possible that Y-NG and Y-WG had other adsorption sites responsible for multilayer adsorption rather than the zeolite phase. Thus, Y-NG and Y-RG were analyzed by several techniques in comparison with Y-WG to understand their adsorption behaviors.

Although Y-NG had the highest adsorption capacity, the conclusion from one point was not firm. Consequently, the synthesis was repeated twice, as described in Section 2.3 of Section 2. The zeolite characteristics and adsorption results are reported in Section 3.4.

### 3.2. Characterization of the Zeolite Samples

**Figure 2:**
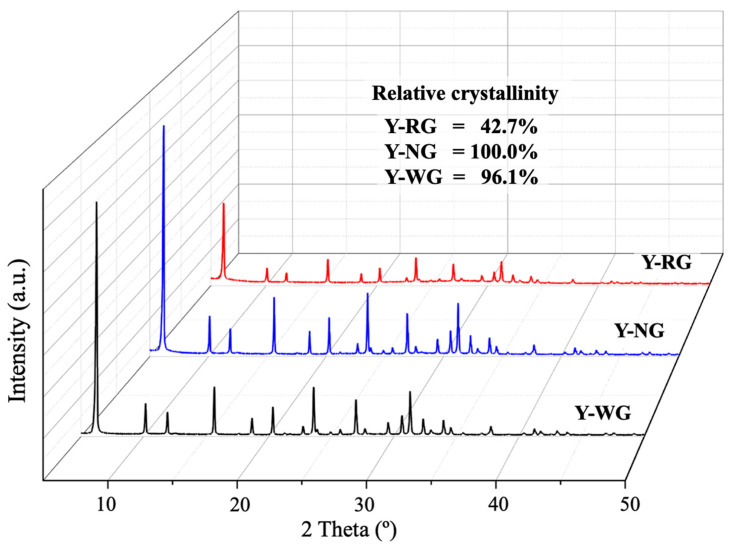
XRD pattern of NaY with refluxing grass (Y-RG) and non-refluxing grass (Y-NG) compared with the original zeolite NaY (Y-WG).

Figure 2 shows the XRD patterns of Y-RG, Y-NG, and Y-WG. All samples exhibit the characteristic peaks of FAU zeolite [1,25]. The zeolite peaks are dominant, providing essentially flat baselines that differ from those of non-refluxed and refluxed grass (Figure S3a in the Supplementary Materials [26]. The relative crystallinity of each sample was determined by comparing the total peak areas of the nine most substantial peaks relative to the sample with the highest total area. The crystallinity of Y-NG was slightly higher than Y-WG and significantly higher than Y-RG. These results indicate that the addition of non-refluxed cogon grass could improve the crystallinity of NaY, whereas the addition of refluxed grass has the opposite effect. When cogon grass is added to the zeolite synthesis, lignin, under alkaline conditions, undergoes degradation, forming small organic molecules [27,28]. The FTIR spectra of non-refluxed grass reveal a prominent peak corresponding to the hydroxyl groups of carbohydrate molecules (refer to Figure S3b and Table S3 in the Supplementary Materials [29,30]). Those groups could affect the zeolite synthesis process.

Y-RG exhibits the lowest crystallinity, indicating that the refluxed grass has an adverse effect on the crystallization process. The reflux process causes the degradation of the grass as depicted in Figure S4. According to Figure S3b, the FTIR spectra confirmed the disappearance of the β-glycosidic linkage [29,30,31]. During reflux, partial complex carbohydrates in cogon grass, such as cellulose and hemicellulose, undergo hydrolysis into simpler sugars like glucose and fructose. Those compounds were subsequently removed by washing them with DI water. Consequently, the remaining fibers possess fewer functional groups, which could affect zeolite synthesis.

Regarding paraquat adsorption, the adsorption behaviors of Y-WG and Y-NG were similar at initial concentrations of 100 to 750 mg/L. The similarity might be due to similar crystallinity since paraquat adsorption occurs in the zeolite cavities [2,24].

**Figure 3:**
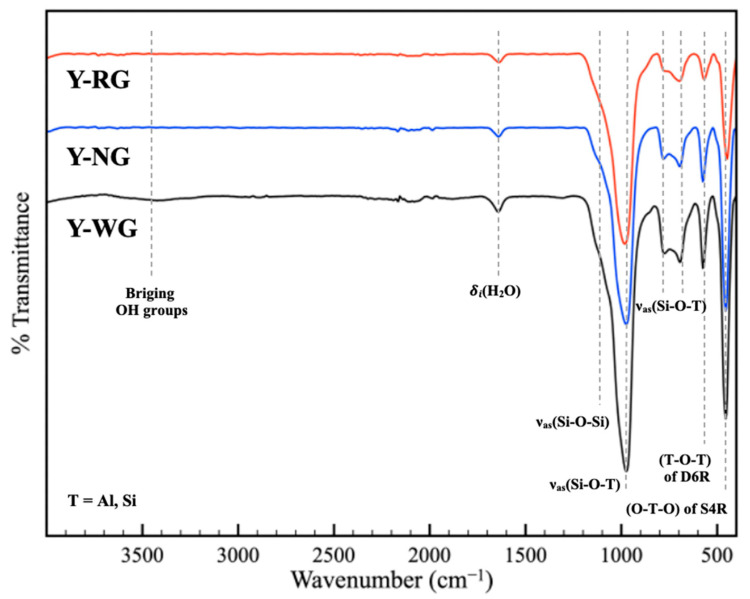
FTIR spectra of Y-WG, Y-NG, and Y-RG.

Figure 3 shows the FTIR spectra of Y-WG, Y-NG, and Y-RG. The spectra of all samples exhibit peaks at similar positions. The peaks originating from internal tetrahedra were assigned as follows [32]: 1130 cm^−1^, asymmetrical stretch (ν_asym_) of Si-O-Si; 695 cm^−1^, symmetrical stretch (ν_sym_) of O-T-O; and 454 cm^−1^, T-O bend. The peaks from the external linkages were assigned as follows: 571 cm^−1^ double-ring vibration; and 1130 cm^−1^ and 772 cm^−1^, asymmetrical and symmetrical stretches of O-T-O, respectively. It is worth noting that some peaks in the Y-RG spectrum exhibit lower intensity than others, such as the peak at 571 cm^−1^ compared to the peak at 695 cm^−1^. This observation aligns with the XRD results, indicating that Y-RG possesses the lowest crystallinity [32].

The peaks originating from hydroxyl groups were identified as follows [32]: the asymmetrical stretch at 3500 cm^−1^ and the deformation band of adsorbed water at 1640 cm^−1^. Among the samples, Y-WG exhibited a stronger intensity in the first band than Y-NG and Y-RG, indicating that Y-WG possessed a higher degree of hydrophilicity.

**Figure 4:**
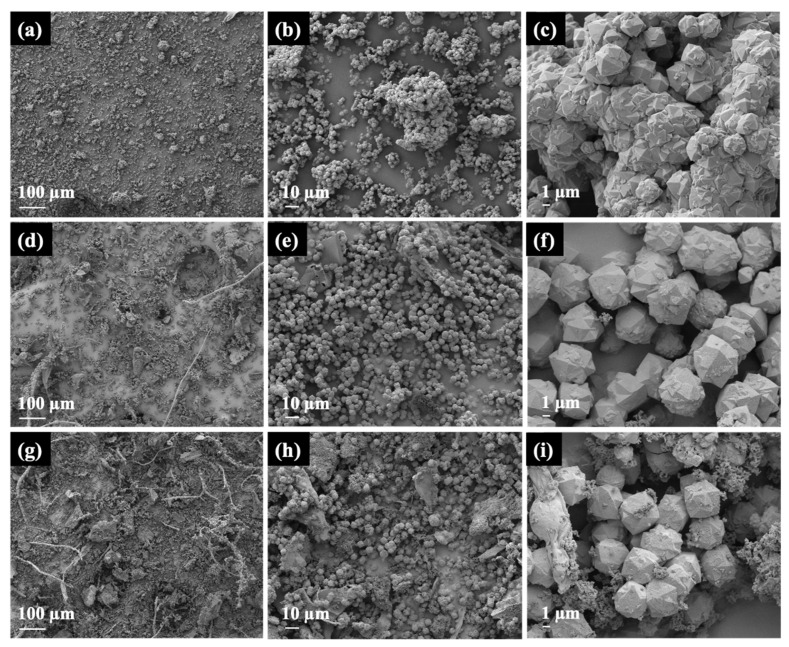
SEM images and EDX spectrum of Y-WG (**a**–**c**), Y-NG (**d**–**f**), and Y-RG (**g**–**i**).

**Figure 5:**
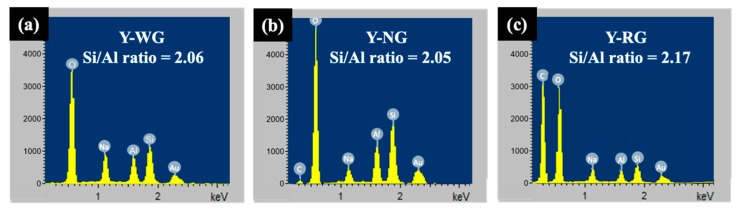
EDX spectrum of Y-WG (**a**), Y-NG (**b**), and Y-RG (**c**).

Figure 4 presents the morphologies of NaY samples obtained from FE-SEM/EDX at different magnifications. In the case of Y-WG, the sample exhibits crystals with particle sizes of approximately 2 µm (Figure 4a). These crystals tend to aggregate, forming particles of varying sizes. Upon closer inspection (Figure 4b,c), it becomes evident that the aggregate comprises polycrystals of different sizes, suggesting a rapid crystallization process. The Si/Al ratio of Y-WG, as determined by EDX, was 2.06 (Figure 5).

The SEM image of Y-NG (Figure 4d) shows particles of zeolite and some fiber, likely from cogon grass. The images at higher magnification (Figure 4e,f) display the zeolite crystals with a uniform size of approximately 5 μm. The images confirm that the addition of cogon grass could prevent the aggregation of zeolite crystals. The Si/Al ratio of Y-NG was 2.05 (Figure 5). Cogon grass contains a low percentage of silica, which did not have much effect on the Si/Al ratio of the zeolite. This sample also contains a small amount of carbon. The aggregation prevention to produce a uniform size distribution was reported in the synthesis of zeolite RHO using methylcellulose as the space-confinement additive [33].

The SEM images of Y-RG in Figure 4g–i show a mixed phase, including fibers, sponge-like materials, and zeolites. The majority of the zeolite phase was polycrystals with a diameter of approximately 5 μm and some woolball-like particles. This sample has a Si/Al ratio of 2.17 and contains more carbon than Y-NG (Figure 5).

Regarding paraquat adsorption, both Y-NG and Y-RG seemed to have multilayer adsorption at the initial concentration of 1000 mg/L. It is possible that multilayer adsorption takes place on carbon compound residues or amorphous phases in Y-NG and Y-RG.

**Figure 6:**
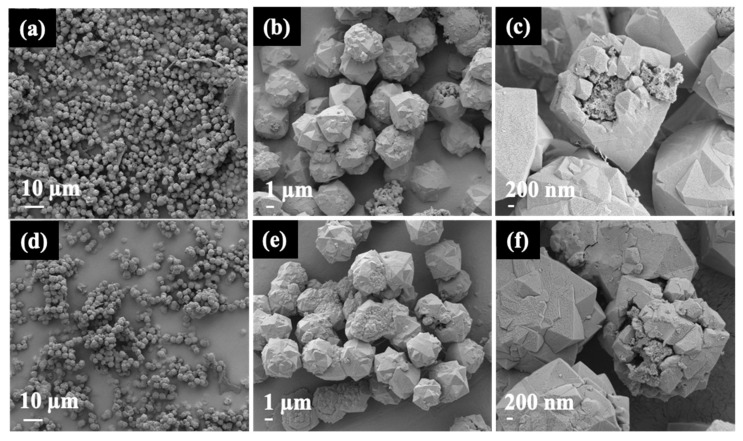
SEM images and EDX spectrum of Y-NG (**a**–**c**) and calcined Y-NG (**d**–**f**).

**Figure 7:**
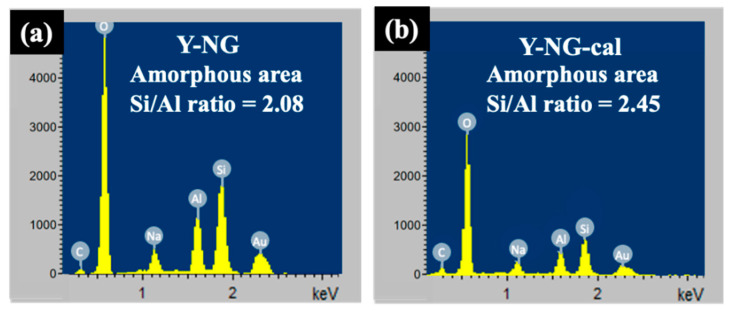
EDX spectrum of the sponge-like amorphous core of Y-NG: before (**a**) and after calcination (**b**).

Figure 6 provides additional SEM images of Y-NG with different magnifications, revealing the presence of crystals and sponge-like particles in the sample. Notably, a few crystals exhibited an open structure (Figure 6b,c), exposing sponge-like materials both inside and outside the crystal. Through EDX point analysis, it was determined that the sponge-like particle is composed of an aluminosilicate material with a Si/Al ratio of 2.08 (Figure 7a). Additionally, carbon was detected in the analysis. The presence of these open crystals suggests incomplete crystallization and offers valuable insights into the mechanism of crystal growth.

To understand the composition of Y-NG, the sample was subjected to calcination at 500 °C for 3 h and investigated using SEM/EDX point analysis. The results are presented in Figure 7b. The presence of open crystals was still observed. Furthermore, Figure 6e illustrates the aggregation of small crystals, leading to the formation of larger particles. The carbon content decreased significantly from 7.1% to 2.8% after calcination (refer to Table S4 in the Supplementary Materials [33,34,35,36,37,38]). The point analysis further confirms that the sponge-like particles consist of aluminosilicate material.

**Figure 8:**
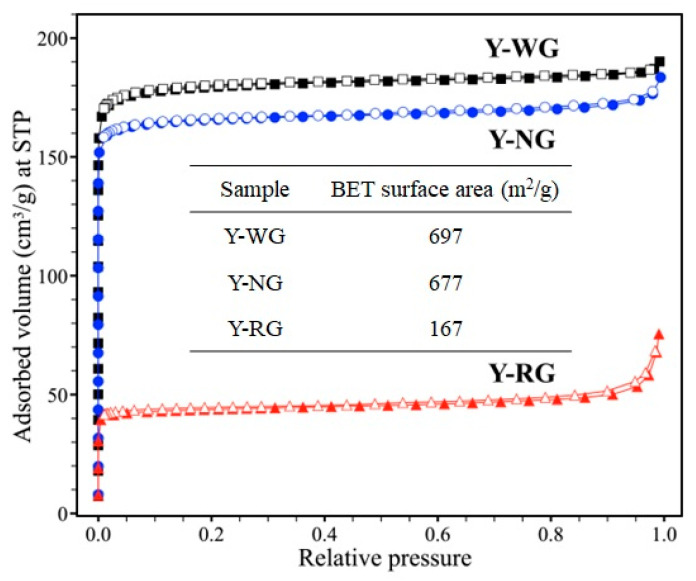
Nitrogen adsorption–desorption isotherm and textural properties of Y-WG, Y-NG, and Y-RG; filled symbols = adsorption, and hollow symbols = desorption. Micropore volume was calculated by the *t*-plot method.

Figure 8 presents the nitrogen adsorption-desorption isotherm for the NaY samples. All samples exhibit a type I isotherm according to the IUPAC classification, which is a characteristic behavior of microporous materials [39]. At low relative pressure (P/P_0_ = 0.0–0.1), the adsorbed volume increases rapidly due to adsorption in the micropores, reaching a plateau as monolayer adsorption occurs. The surface area and pore volume of Y-NG are slightly smaller than those of Y-WG, potentially attributed to the presence of amorphous particles. Y-RG shows the lowest surface area and pore volume, primarily due to a significant amount of the amorphous phase. The findings from the nitrogen adsorption–desorption analysis align with the results obtained from the XRD and SEM observations.

From the presented results, the addition of cogon grass to the synthesis of NaY could prevent the aggregation of zeolite crystals. Non-refluxed grass gave NaY a higher crystallinity and surface area than refluxed grass.

### 3.3. Characterization of Y-NG with Various Grass Content

**Figure 9:**
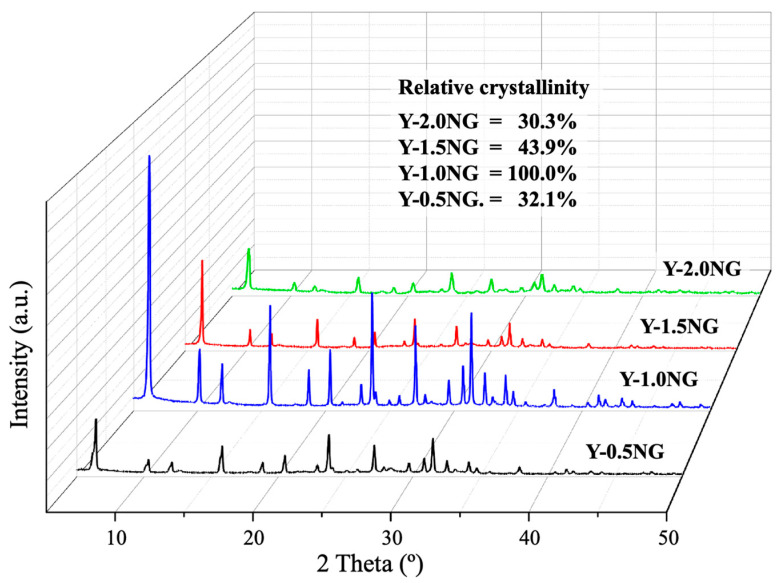
XRD patterns of Y-NG synthesized with various grass content.

Figure 9 displays the XRD pattern of NaY synthesized with different amounts of non-refluxed grass. The characteristic peaks of zeolite NaY were observed in all samples. Y-1.0NG exhibited the highest crystallinity, followed by Y-1.5NG, Y-0.5NG, and Y-2.0NG.

**Figure 10:**
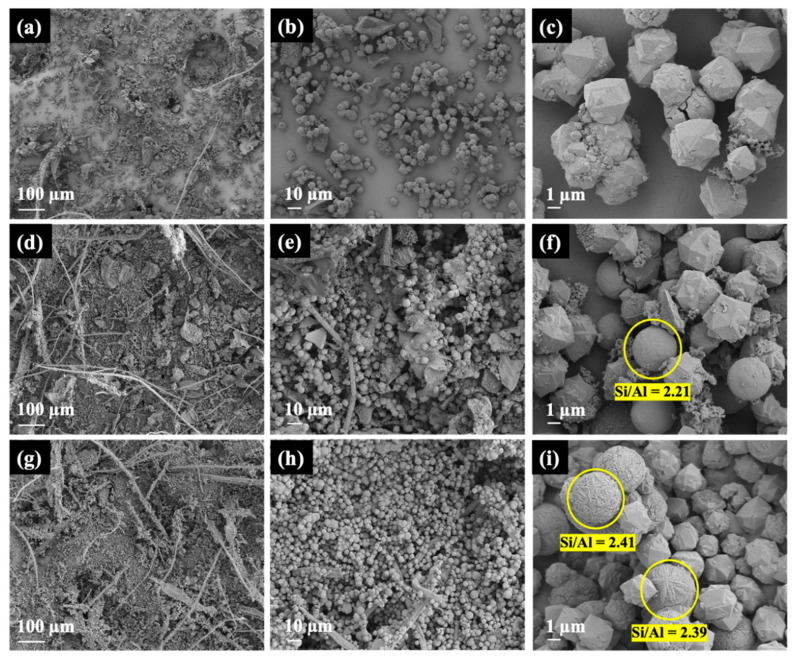
SEM images of Y-0.5NG (**a**–**c**), Y-1.5NG (**d**–**f**), and Y-2.0NG (**g**–**i**).

**Figure 11:**
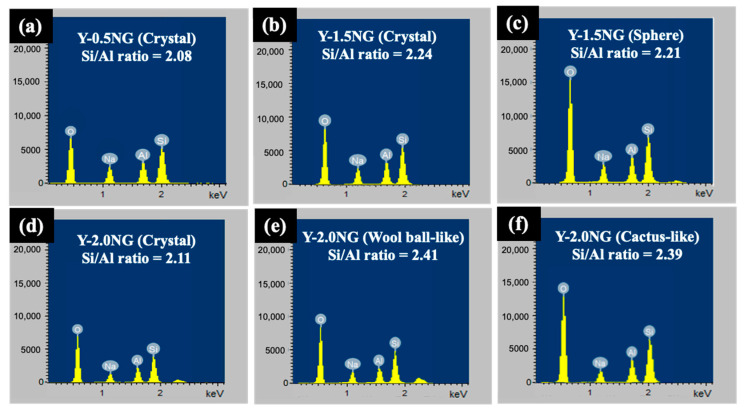
EDS spectra of Y-0.5NG (**a**), Y-1.5NG (**b**,**c**), and Y-2.0NG (**d**–**f**).

Figure 10 presents the SEM images of Y-0.5NG, Y-1.5NG, and Y-2.0NG at various magnifications. The images of Y-0.5NG (Figure 10a–c) reveal the presence of crystals and round particles with an approximate diameter of 5 μm and amorphous particles. These round-shaped and amorphous particles could contribute to the lower intensity in the XRD pattern. The Si/Al ratio of Y-0.5NG from SEM-EDS was 2.08 (Figure 11). The images of Y-1.5NG (Figure 10d–f) also show crystals, round particles, and sponge-like phases. However, the Si/Al ratios of the crystal and round particles were similar (namely, 2.24 and 2.21). Lastly, the mages of Y-2.0NG (Figure 10g–i) show particles of polycrystals and ball wool-like, and cactus-like round particles. The Si/Al ratios of these particles were as follows: 2.11 (crystals), 2.41 (wool ball-like shape), and 2.39 (cactus-like shape). Their EDX spectra are shown in Figure 11. The results above clearly indicate that the amount of grass has a significant impact on the synthesis of zeolite NaY. During the base hydrolysis process, lignocellulose undergoes decomposition, yielding sugar, alcohol, and other molecules [17,40]. The increased sugar content can reduce the aluminum (Al) content within the zeolite structure. Sugar molecules tend to preferentially bind with silicate precursors rather than aluminate precursors, leading to a higher silicon-to-aluminum (Si/Al) ratio [15,41]. Concurrently, the presence of aluminum slows down the growth of crystal edges. The optimal amount of grass in the synthesis of zeolite NaY is 1.0 g.

**Figure 12:**
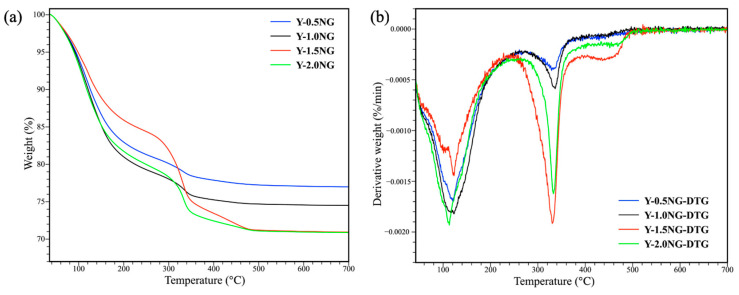
TGA (**a**) and DTG (**b**) curves of Y-NG with various grass content.

Figure 12 illustrates the thermogravimetric analysis (TGA) and derivative thermogravimetry (DTG) plots of Y-NG obtained from different amounts of grass. The TGA plots exhibit weight losses in three distinct regions. The weight loss in the temperature ranges up to 250 °C corresponds to the desorption of water [42]. The weight loss at higher temperatures is due to the decomposition of residual grass fibers. The pyrolysis process involving the breakdown of cellulose, hemicellulose, and lignin occurs in the 315–400 °C temperature range [43]. Finally, the combustion of the complex structure and the formation of char take place above 400 °C on the Y-1.5NG and Y-2.0NG samples [44]. 

### 3.4. Characterization and Paraquat Adsorption of Non-Calcined and Calcined Y-NG

**Figure 13:**
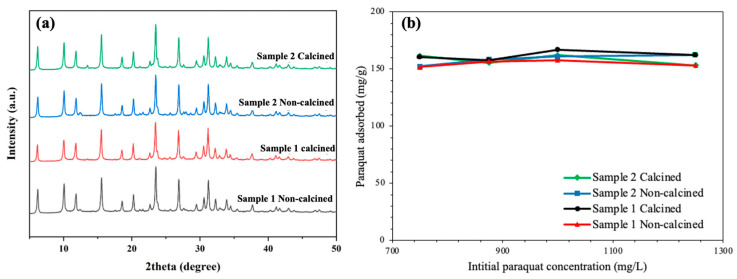
XRD patterns of non-calcined and calcined YNG (**a**) and paraquat adsorption of Y-NG and Y-WG from various initial concentrations (**b**).

Figure 13a displays the XRD patterns of non-calcined and calcined Y-NG synthesized with 1.0 g of non-refluxed grass. The characteristic peaks of zeolite NaY were observed in all samples. Note that it was observed before from the same NaY sample that the intensity of the first peak from the Bruker D2 diffractometer is lower than that of the Bruker D8. The weights of calcined samples 1 and 2 were 10.7% and 8.9%, which are less than the non-calcined samples due to the loss of water and organic content. The SEM images of non-calcined and calcined samples show good dispersion and a Si/Al ratio of 1.9. The paraquat adsorption of Y-NG samples reached a plateau at higher initial concentrations as shown in Figure 13b, indicating that a monolayer was formed. The adsorption behavior was similar to the report by Keawkumay et al. [3]. From these results, one can conclude that the remaining organic content in the zeolite samples and calcination did not affect the adsorption behavior of zeolite NaY.

## 4. Conclusions

Non-refluxed and refluxed cogon grass (NG and RG) were added to the synthesis of zeolite NaY to produce Y-NG and Y-RG. NaY was also synthesized without grass addition (Y-WG) for comparison. All samples adsorbed the paraquat according to the Langmuir model. The adsorption mainly takes place on zeolite. Y-NG has comparable crystallinity and surface area to Y-WG and better particle dispersion. In contrast, Y-RG displayed the lowest crystallinity, surface area, and paraquat adsorption capacity. This work demonstrates the potential of cogon grass to produce zeolite with uniform crystal size and good dispersion.

Future research directions can focus on a deeper understanding of the role of compounds generated from cogon grass during zeolite synthesis and finding unique applications of the uniform-sized zeolite. This study exemplifies the potential of cogon grass as an abundant and natural resource for developing sustainable and environmentally friendly materials in adsorption and catalysis applications.

## Figures and Tables

**Table 1:** The parameters of paraquat adsorption fit with the Langmuir and Freundlich models.

Sample	Langmuir Model			Freundlich Model		
	*q_m_* (mg/g)	*K_L_* (L/mg)	R^2^	*K_F_* (mg/g)	*n*	R^2^
Y-WG	150.8 ± 1.3	0.3350	0.9997	46.5	4.890	0.8648
Y-NG	178.0 ± 1.8	0.0522	0.9915	44.5	4.410	0.9071
Y-RG	80.0 ± 8.0	0.1434	0.9814	5.02	15.39	0.9274

## Data Availability

Data are contained within the article and Supplementary Materials.

## Supplementary Materials

The following supporting information can be downloaded at: https://www.mdpi.com/article/10.3390/ma16237330/s1. Figure S1: Calibration curve of paraquat solution for UV-Vis determination; Table S1: The equilibrium concentration (*C_e_*), equilibrium capacity of paraquat adsorption (*q_e_*), and *C_e_*/*q_e_* value of zeolite NaY samples at concentrations of 100 to 1000 mg/L; Figure S2: Plot of Langmuir (**a**) and Freundlich adsorption isotherms; Table S2: Paraquat adsorption of zeolite NaY samples; Figure S3: XRD pattern (a) and FTIR spectra (b) of untreated and chemical-modified grass; Table S3: Peak assignments of functional groups of samples; Figure S4: SEM image of the cogon grass in natural (NG), acid-treated grass (RG), and alkali-soaked samples (NGS and RGS). Table S4: Chemical compositions of the internal zeolite NaY particles. Ref. [31] can be found in Supplementary Materials.
