# Supplementary material for: The Synthesis of Well-Dispersed and Uniform-Sized Zeolite NaY by Adding Non-Refluxed and Acid-Refluxed Cogon Grass
Source: Materials (Basel). 2023 Nov 24;16(23):7330. doi: 10.3390/ma16237330 (PMC10707157; doi:10.3390/ma16237330)
Supplement: Supplementary file 1 [file materials-16-07330-s001.zip › materials-2668561-supplementary.pdf]

Supplementary Materials

The Synthesis of Well-Dispersed and Uniform-Sized Zeolite NaY by Adding Non-Refluxed and Acid-Refluxed Cogon Grass

Pakawan Sereerattanakorn [1], Pimwipa Tayraukham [1], Nattawut Osakoo [1,2], Panot Krukkratoke [1], Chalermpan Keawkumay [1,2], Jatuporn Wittayakun [1,*], Nichakorn Pornnongsan [1], Krittanun Deekamwong [1,2] and Sanchai Prayoonpokarach [1,*]

[1] School of Chemistry, Institute of Science, Suranaree University of Technology, Nakhon Ratchasima 30000, Thailand; pakawan.se@gmail.com (P.S.); pimwipatrk@gmail.com (P.T.); nattawut.o@sut.ac.th (N.O.); d6400040@g.sut.ac.th (P.K.); chalermpan@sut.ac.th (C.K.); nichakornbubbee@gmail.com (N.P.); krittanun.d.aa@sut.ac.th (K.D.)

[2] Institute of Research and Development, Suranaree University of Technology, Nakhon Ratchasima 30000, Thailand

* Correspondence: jatuporn@sut.ac.th (J.W.); sanchaip@sut.ac.th (S.P.)

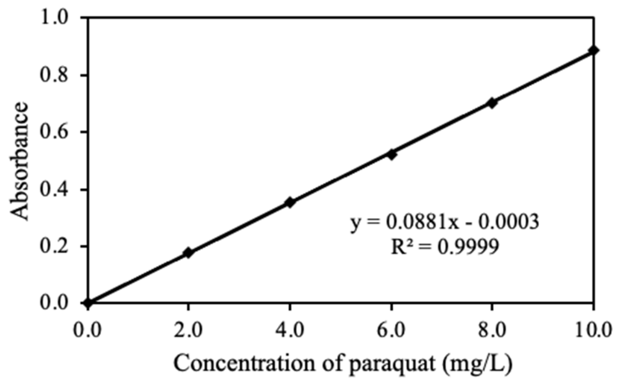

Figure S1. Calibration curve of paraquat solution for UV-Vis determination.

Table S1. The equilibrium concentration (C_e), equilibrium capacity of paraquat adsorption (q_e), and C_e/q_e value of zeolite NaY samples at concentration 100 to 1000 mg/L.

Concentrations of paraquat solution		Sample name/Experiment								
		Y-WG			Y-NG			Y-RG		
		1st	2nd	3rd	1st	2nd	3rd	1st	2nd	3rd
100 ppm	C_e	1.33	1.29	1.24	1.33	1.38	1.35	1.89	2.06	1.92
	q_e	38.76	38.04	38.50	38.02	38.75	38.84	36.88	37.96	38.01
	C_e/q_e	0.035	0.034	0.032	0.035	0.036	0.035	0.051	0.054	0.051
250 ppm	C_e	7.74	7.71	6.57	8.89	6.55	7.81	69.87	69.24	68.14
	q_e	94.08	95.77	93.09	94.92	95.85	93.87	96.74	96.37	96.76
	C_e/q_e	0.082	0.081	0.071	0.094	0.068	0.083	0.014	0.014	0.014
500 ppm	C_e	131.58	142.27	131.18	158.79	103.74	157.92	304.21	307.91	307.85
	q_e	143.63	139.23	144.35	132.25	155.09	132.59	77.39	74.60	74.05
	C_e/q_e	0.916	1.021	0.909	1.201	0.669	1.191	3.931	4.128	4.158
750 ppm	C_e	369.84	365.59	355.51	352.77	352.95	383.28	549.14	567.59	571.72
	q_e	150.26	152.24	152.61	156.70	153.30	144.95	146.46	149.73	150.31
	C_e/q_e	2.461	2.401	2.329	2.251	2.302	2.644	6.971	8.106	8.209
1000 ppm	C_e	624.34	609.21	617.46	530.57	531.94	529.65	758.13	805.94	798.14
	q_e	146.46	149.73	150.31	181.60	185.00	186.65	93.75	75.81	79.63
	C_e/q_e	4.263	4.069	4.108	2.921	2.875	2.838	8.087	10.632	10.023

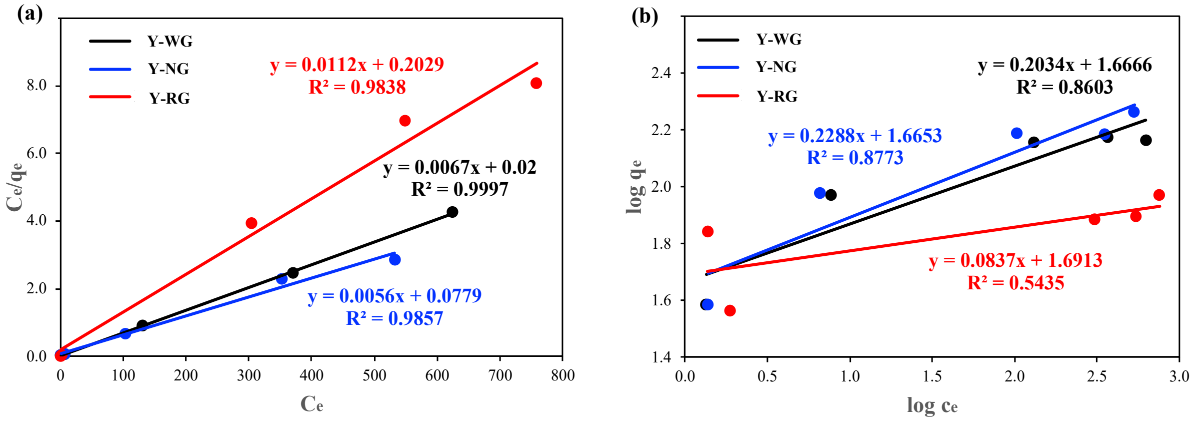

Figure S2. Plot of Langmuir (a) and Freundlich adsorption isotherm (b).

Table S2. Paraquat adsorption of zeolite NaY samples.

Sample	Maximum adsorption capacity of paraquat, q_m (mg/g)			
	1st trial	2nd trial	3rd trial	Means
Y-WG	149.25	151.52	151.52	150.76 ± 1.30
Y-NG	178.57	178.57	175.44	177.53 ± 1.81
Y-RG	89.29	74.07	76.92	80.09 ± 8.09

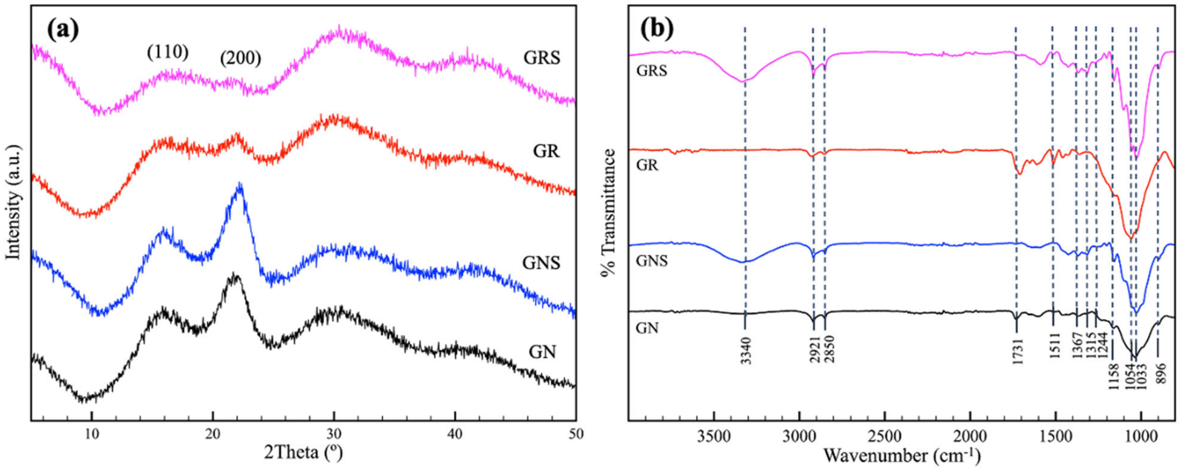

Figure S3. XRD pattern (a) and FTIR spectra (b) of untreated and chemical-modified grass.

Figure S3 presents XRD pattern and FTIR spectra of cogon grass in its natural state and after treatment. The treated grass exhibits notable changes compared to the raw grass, primarily attributed to the disruption of crystalline cellulose, hemicellulose, and lignin components within the lignocellulosic biomass. As a result, the treated grass becomes more susceptible to cellulose presence [1].

Following the acid treatment, a distinctive peak at 896 cm^{-1}, corresponding to the β-glycosidic linkage between glucose units in cellulose, becomes absent in the RG sample. This absence suggests the cleavage of bonds and decomposition of cellulose, resulting in the conversion of a portion of cellulose into sugar units. Additionally, Figure 5a illustrates the characteristic peaks associated with crystalline cellulose at 15.5° and 22°, corresponding to the 110 and 200 planes, respectively [2]. These findings indicate a significant reduction in the crystallinity of cellulose following the acid treatment.

Moreover, the FT-IR spectra of the samples subjected to alkaline hydrolysis are presented. The spectral regions at 1260, 1314, and 1369 cm cm^{-1} correspond to hemicellulose, which is obtained through alkali hydrolysis [3]. The signals attributed to the aromatic vibrations of lignin at 1511 and 1729 cm^{-1} disappear in the NGS and RGS samples, indicating the dissolution of lignin in the alkaline solution. Notably, a broad band emerges at 3340 cm cm^{-1}, indicating an increase in the cellulose fraction. Additionally, the XRD pattern shows no discernible difference between the untreated and base-treated samples, suggesting that the crystalline cellulose remains unaffected by the base degradation. It is worth mentioning that the chemical treatment significantly reduces the complexity of the lignocellulosic fiber.

Table S3. Peak assignments of functional groups of samples.

Wavenumber (cm^{-1})	Assignment	Reference
3340	O-H group stretching of cellulose	[2,5]
2921	C-H stretching in lignocellulose	
2850	Methyl and methylene groups of lignin	
1731	C=O acetyl group or carbonyl group of hemicellulose and lignin unit	[6,7]
1511	Aromatic stretching of lignin	
1367, 1315, 1244	C-O and C-H bending of hemicellulose	[3,8]
1158	C-O-C glycosidic bond of cellulose and hemicellulose	
1054	C-O in C-O-C ring skeletal vibration of cellulose and lignin	[4,5]
1033	C-O-H stretching of alcohol of cellulose and lignin	
896	β-glycosidic linkage between glucose units of cellulose	[9]

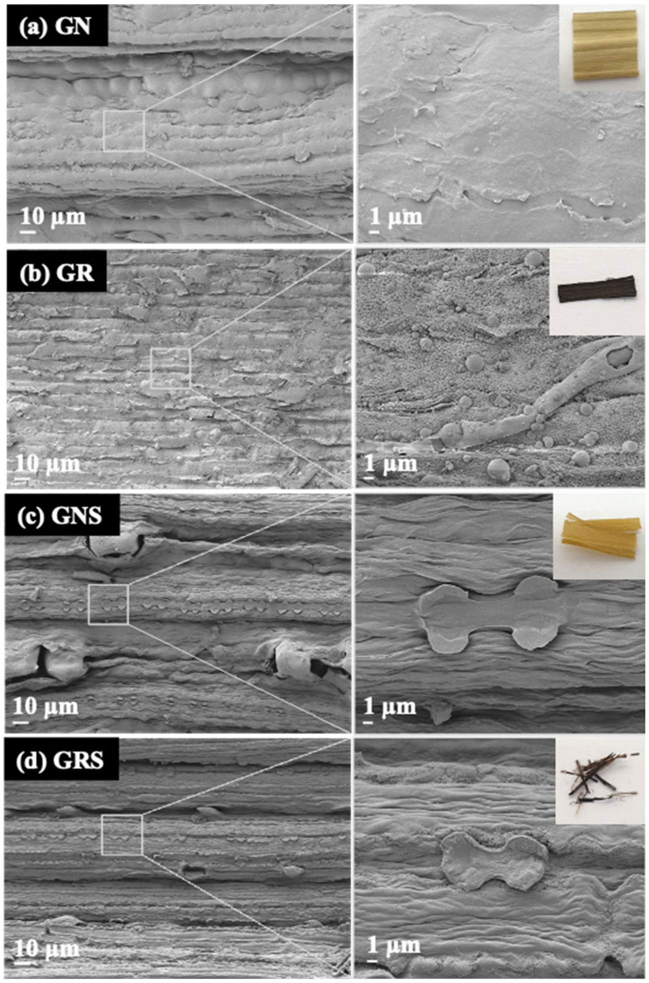

Figure S4. SEM image of the cogon grass in natural (NG), acid-treated grass (RG), and alkali-soaked samples (NGS and RGS).

Figure S4 depicts the untreated grass and its appearance after acid treatment. The untreated grass exhibits a smooth surface with a continuous fiber structure (Figure S4a). However, upon HCl treatment, noticeable disruptions in the fiber structure occur, resulting in a rough surface (Figure S4b), which is consistent with findings reported by Zarib and coworkers [10]. Furthermore, after NaOH soaking, the surface of the grass becomes even more distorted (Figure S4c-d). The surface of the acid-treated grass (RGS) exhibits higher abrasion compared to the untreated samples.

Table S4. Chemical compositions of the internal zeolite NaY particles.

Sample name	Compositions (%)				
	C	O	Na	Al	Si
Y-NG	7.14 ± 3.82	56.63 ± 2.75	2.48 ± 0.62	10.97 ± 0.65	22.77 ± 0.59
Y-NG-cal	2.78 ± 0.54	54.00 ± 0.57	4.09 ± 1.54	11.04 ± 0.06	27.00 ± 0.69

References

1. Amin, F. R., Khalid, H., Zhang, H., Rahman, S. U., Zhang, R., Liu, G., Chen, C. Pretreatment methods of lignocellulosic biomass for anaerobic digestion. *AMB Express* **2017,** 7, 1, 72.
2. Kandhola, G., Djioleu, A., Rajan, K., Labbé, N., Sakon, J., Carrier, D. J., Kim, J.-W. Maximizing production of cellulose nanocrystals and nanofibers from pre-extracted loblolly pine kraft pulp: a response surface approach. *Bioresources and Bioprocessing* **2020,** 7, 1,
3. Xu, F., Sun, J. X., Geng, Z. C., Liu, C. F., Ren, J. L., Sun, R. C., Fowler, P., Baird, M. S. Comparative study of water-soluble and alkali-soluble hemicelluloses from perennial ryegrass leaves (Lolium peree). *Carbohydrate Polymers* **2007,** 67, 1, 56-65.
4. Lopes, J. d. O., Garcia, R. A., Souza, N. D. d. Infrared spectroscopy of the surface of thermally-modified teak juvenile wood. *Maderas. Ciencia y tecnología* **2018**, 20, 4, 737-746.
5. Kundu, C., Samudrala, S. P., Kibria, M. A., Bhattacharya, S. One-step peracetic acid pretreatment of hardwood and softwood biomass for platform chemicals production. *Sci Rep* **2021,** 11, 1, 11183.
6. Nandanwar, R., Chaudhari, A., Ekhe, J. Nitrobenzene oxidation for isolation of value added products from industrial waste lignin. *J. Chem. Biol. Phys. Sci* **2016,** 6, 501-513.
7. Khalid, F. E., Ahmad, S. A., Zakaria, N. N., Shaharuddin, N. A., Sabri, S., Azmi, A. A., Khalil, K. A., Verasoundarapandian, G., Gomez-Fuentes, C., Zulkharnain, A. Application of Cogon Grass (Imperata cylindrica) as Biosorbent in Diesel-Filter System for Oil Spill Removal. *Agronomy* **2021,** 11, 11,
8. Yuan, Z., Kapu, N. S., Martinez, D. M. An eco-friendly scheme to eliminate silica problems during bamboo biomass fractionation. *Nordic Pulp & Paper Research Journal* **2017,** 32, 1, 4-13.
9. Haque, M. A., Barman, D. N., Kim, M. K., Yun, H. D., Cho, K. M. Cogon grass (Imperata cylindrica), a potential biomass candidate for bioethanol: cell wall structural changes enhancing hydrolysis in a mild alkali pretreatment regime. *J Sci Food Agric* **2016,** 96, 5, 1790-1797.
10. Zarib, N., Abdullah, S., Jusri, N., Effect of acid treatment on extraction of silica from cogon grass by using $C_6H_8O_7$ and HCl Acid. In *IOP Conference Series: Materials Science and Engineering*, IOP Publishing **2020**, 834, 012067.
